# Consequences of variation in foraging success among predators on numerical response

**DOI:** 10.1002/ece3.772

**Published:** 2013-09-17

**Authors:** Toshinori Okuyama

**Affiliations:** Department of Entomology, National Taiwan UniversityTaipei, Taiwan

**Keywords:** Fecundity, functional response, individual variation, Jensen's inequality, numerical response.

## Abstract

The relationship between foraging success and reproduction is commonly assumed to be linear in theoretical investigations. Although the exact relationship (e.g., linear or nonlinear) does not influence qualitative conclusions of models under some assumptions, an inclusion of individual behavioral variation can make it otherwise due to Jensen's inequality. In particular, a mechanism that stabilizes food web dynamics is generated when two conditions are satisfied: (1) the reproduction of predators experiences diminishing returns from foraging success (i.e., concave down relationship between foraging success and reproduction) and (2) foraging success variation among predator individuals increases with the predator density. However, empirical results that confirm these conditions are scarce. This study describes the mechanism as a hypothesis for stability and discusses some important considerations for empirical verifications of the mechanism.

## Introduction

Birth and death are two important processes of population and community dynamics. Functional response directly describes a part of death processes of prey by describing the relationship between an individual predator's rate of consuming prey and environmental variables such as the prey and predator densities. Similarly, numerical response describes the relationship between an individual predator's fitness (e.g., birth/reproduction and death rates) and environmental variables. Because the fitness of a predator depends on its foraging success, functional response and numerical response are closely related. However, although much attention has been given to describing functional response (Oaten and Murdoch 1975; Abrams 1982; Abrams and Ginzburg 2000; Arditi and Ginzburg 2012), little attention has been given to the relationship between functional and numerical responses and their consequences in ecological dynamics (but see Crawley 1975; Abrams 2000, 2002).

For a given functional response *f* (e.g., *f* = *aN/*(1 + *ahN*) for a type II functional response where *a* and *h* are the attack rate and handling time parameters, respectively), one of the most common ways to relate the functional response and numerical response is *bf* where *b* is the conversion parameter that converts consumption to reproduction. In other words, *bf* describes the reproduction part of numerical response and assumes that the birth rate of a predator linearly increases with the amount of prey taken by the predator. Although this convention is commonly used, any organisms must experience some upper limit in their reproduction rates (discussed further in *Discussion*).

**Figure 1:**
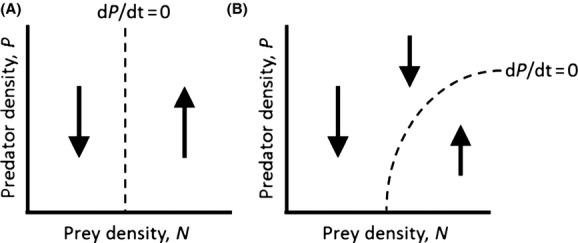
Phase-plane diagrams for the predator population. The dashed line is the predator's isocline (i.e., d*P*/d*t* = 0). The arrows show whether the predator population size is increasing (upward arrow: d*P*/d*t >* 0) or decreasing (downward arrow: d*P*/d*t <* 0). In (A), the predator isocline is independent of the predator density. In (B), the predator density influences the isocline.

There may be a variety of reasons why the linear relationship is conventionally accepted. Among them, the main reason probably is because the linear assumption does not affect model predictions in important ways even if it were wrong. Here, I give an example using the Rosenzweig–MacArthur model (Rosenzweig and MacArthur 1963) that describes the dynamics of the prey density *N* and the predator density *P* as,


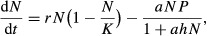
(1)


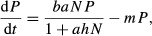
(2)

where *r*, *K*, and *m* are the intrinsic rate of increase of the prey, carrying capacity of the prey, and density-independent mortality rate of the predator, respectively. Because the interest here is the numerical response, we focus on the predator equation (2). In this model, the predator isocline (d*P/*d*t* = 0) is,


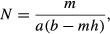
(3)

which corresponds with Figure 1A. In other words, the isocline is independent of the predator density.

Suppose we consider a nonlinear relationship between reproduction and consumption phenomenologically as,


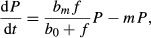
(4)

where *b*_*m*_ and *b*_0_ are the parameters that characterize the nonlinear relationship, and *f* = *aN*/(1 + *ahN*). The predator isocline based on equation (4) is,


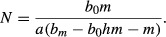
(5)

Thus, the predator isocline is still independent of the predator density (Fig. 1A). This result (i.e., predator-independent isocline) is not specific to the nonlinear relationship used (*b*_*m*_*f/*(*b*_0_ + *f*)) and holds for any other nonlinear relationships. Given this, it is understandable that not much attention has been given to the exact relationship between functional response and numerical response.

The purpose of this study is to show that the above result (i.e., nonlinearity does not affect qualitative results) breaks down in important ways when we start considering a fundamental factor, that is, the individual variation in foraging success among predator individuals. For example, in a model, *fT* predicts the number of prey consumed by a predator in the duration *T* (if the densities do not change). However, this prediction is on average, and the actual number of prey consumed by predator individuals is variable around the average. Conventional models (e.g., equations 1 and 2) commonly assume that this type of variation does not affect model prediction although the assumption is not valid (e.g., Okuyama 2008; Bolnick et al. 2011). This study hypothesizes that these two factors (nonlinearity and individual variation) generally stabilize food web dynamics. In the following sections, the stability mechanism is explained and testable predictions are given in order to facilitate the empirical verification of the mechanism.

### Foraging success variation among predators

To illustrate the mechanism in which the relationship between reproduction and foraging success becomes important, a simple example is discussed here. There are four predators (but the argument made here can be generalized to any population sizes). In a given foraging duration, these predators captured 2, 4, 6, and 8 prey (i.e., the average number of prey captured by an individual predator is 5). If a predator can produce *b* offspring if it consumes *b* prey (e.g., a linear relationship between foraging success and reproduction), then the total number of offspring produced by the group of predators is 20 (i.e., 2 + 4 + 6 + 8). This prediction is also the same even when we ignore the individual variation in foraging success. That is, if we consider that each predator captured 5 prey, the predicted total reproductive output is 20 (i.e., 5 + 5 + 5 + 5). In this case, individual variation does not influence the prediction.

However, when the relationship between foraging success and reproduction is not linear, individual variation can influence the predator population's growth rate. For example, suppose there is a maximum number of offspring for a predator, and it is five in this example (i.e., no matter how many prey a predator consumes, it can only reproduce at most/maximum five offspring). Then in reality, the total number of offspring is 16 (i.e., 2 + 4 + 5 + 5). However, if we ignore individual variation and assume that each predator captured five prey, the model will predict 20 offspring (i.e., 5 + 5 + 5 + 5). Thus, the relationship between foraging success and reproduction matters when we start considering individual variation.

More generally, Jensen's inequality states that


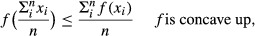
(6)


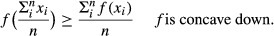
(7)

In the above example, *f*(*x*) = *x* if *x <* 5 and *f*(*x*) = 5 if *x* ≥ 5 (i.e., *f* is concave down), resulting in *f*((2 + 4 + 6 + 8)/4) = 5 *>* 4 = (*f*(2) + *f*(4) + *f*(6) + *f*(8))/4. This bias does not exist if there is no individual variation (e.g., all individuals captured 5 prey): *f*((5 + 5 + 5 + 5)/4) = (*f*(5) + *f*(5) + *f*(5) + *f*(5))/4.

The effect described here can create a self-limiting effect on the predator population growth. For example, if individual variation in foraging success increases with the predator density and the relationship between foraging success and reproduction is concave down (e.g., equation 4), the predator isocline can change from Fig. 1(A) to Fig. 1(B) even when the functional response (e.g., mean effect) is independent of the predator density. It is easy to show that this change in the isocline generally stabilizes community dynamics (Hastings 1997; Case 2000; Gottelli 2008; McPeek 2012).

### Density-dependent individual variation

Although demonstrating whether individual variation exists is trivial, one of the main issues is that we know little about, for example, how individual variation in foraging success changes with the predator density (and also with other variables). In fact, many empirical functional response studies assume that even mean response is independent of the predator density without having any data (e.g., experimental design does not test multiple predator levels) (Okuyama and Ruyle 2011; Okuyama 2013). Even when empirical studies consider the effect of the predator density (e.g., have multiple levels of the predator density in experimental design), the available data are the total number of prey consumed by the predator population in a given time, and thus, the individual-level data are usually not available (e.g., Kratina et al. 2009).

In the absence of empirical data, theoretical studies may provide some expectations. For example, a simple spatially explicit individual-based model shows that the individual variation increases with the predator density even when the predators do not directly interact with each other (Okuyama 2009). This effect emerges from spatial shadow competition in which a predator's foraging success is influenced by other predators because, for example, prey that were otherwise captured by the focal predator can be intercepted by other predators in the environment. Thus, predators are not truly independent of each other as long as they forage in the common environment.

**Figure 2:**
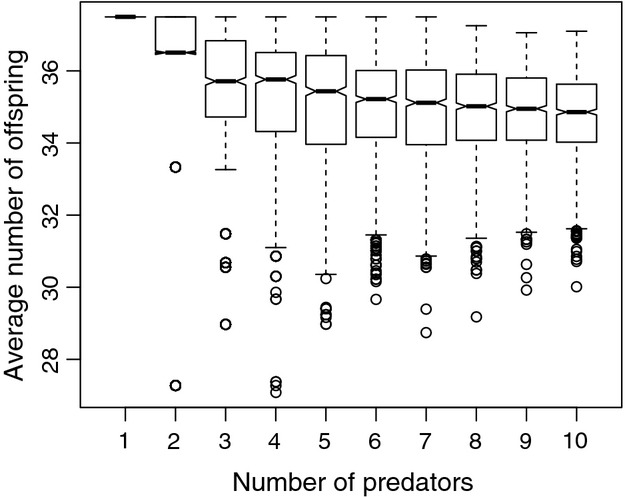
Relationship between the average number of offspring per predator and the number of predators. Boxplot shows the variability in the simulation results based on 1000 simulation runs at each level of the number of predators, *n*. *U* = 3*; β*_m_ = 100*; β*_0_ = 5.

A related phenomenon can be illustrated by a simple simulation model. Suppose there is only one predator, the predator captures three prey (*U* = 3). When there are two predators, six prey are shared by the two predators (*x*_1_ + *x*_2_ = 6) where *x*_i_ is the number of prey consumed by the *i*th predator. Each prey is randomly allocated to the predators so that *x*_1_ and *x*_2_ are variable due to the random process (e.g., *x*_1_ = 1*; x*_2_ = 5; and *x*_1_ = 6*; x*_2_ = 0). When there are three predators, nine prey are allocated to the three predators (*x*_1_ + *x*_2_ + *x*_3_ = 9), and so on. In general, when there are *n* predators, *nU* prey are shared randomly among them so that the average number of prey captured by a predator is always *U* (i.e., *nU*/*n*). Suppose the number of offspring produced by a predator that consumed *x* prey is *B*(*x*) = *β*_m_*x*/(*β*_0_ + *x*) where *β*_m_ and *β*_0_ are the positive-valued parameters that characterize the relationship. Under these assumptions, when there is only one predator, the number of offspring produced by the predator is *B*(3). When there are two predators, *B*(*x*_1_) and *B*(*x*_2_) offspring are produced, and the average number of offspring (*y*-axis in Fig. 2) when there are two predators (*B*(*x*_1_) + *B*(*x*_2_))/2 is variable because *x*_1_ and *x*_2_ can be variable. The average fecundity when there are two predators is generally lower than the fecundity of the predator when there is only one individual (*B*(*x*_1_) + *B*(*x*_2_))/2 *<* *B*(3). In fact, they are the same only when there is no variation in foraging success (*x*_1_ = *x*_2_ = 3): (*B*(*x*_1_) + *B*(*x*_2_))/2 = *B*(3). More generally, variation in foraging success increases with the predator density in the simulation, and thus, the average foraging success decreases with the predator density (Fig. 2). This qualitative pattern is robust to changes in the values of the parameters (*U*, *β*_m_, *β*_0_) in the model. This decrease in the per capita fitness of the predator with the predator density changes the shape of the isocline from Figure 1(A) to Figure 1(B).

## Discussion

In theoretical investigations, whether we use linear or nonlinear relationship between foraging success and reproduction may not affect general conclusions under some assumptions. However, an inclusion of individual variation makes it an important consideration. Given that the relationship between foraging success and reproduction can be nonlinear for a variety of reasons (Crawley 1975) and the individual variation would almost always exist, the mechanism described in this study may play important roles in general ecological dynamics.

It is important to reiterate that the effect described here can be independent of functional response. Although the example used in this study is based on a predator-independent functional response (i.e., type II model), it can operate with any functional response models (including predator-dependent models). Therefore, when examining the relationship between fecundity and the amount of prey eaten, care is needed. For example, in common numerical response studies in which the relationship between fecundity and the prey density (i.e., not the actual amount of prey eaten) is characterized (e.g., Hemptinne et al. 1992; Agarwala and Bardhanroy 1999; Castagnoli and Simoni 1999), the results are confounded with functional response. This is because a nonlinear relationship between reproduction and the prey density can emerge from a nonlinear functional response even if the relationship between reproduction and foraging success is linear. Experiments that directly examine the relationship between fecundity and the amount of resources consumed are needed (but see below for further considerations).

Similarly, one may argue that functional response effectively limits the number of prey that can be eaten by a predator so that it is not necessary to consider reproductive limitations. In fact, some studies show linear relationships between reproduction and the amount of prey consumed (e.g., Matsura and Morooka 1983). However, even in this situation, nonlinear relationships may emerge from the relationship between the body size and the amount of prey eaten (and/or the relationship between body size and fecundity) as larger-bodied individuals tend to reproduce more (Büns and Ratte 1991; Honěk 1993). In other words, a controlled experiment (e.g., controlled for the body size and life stage of study subjects) does not necessarily provide the complete picture. Furthermore, if it takes time to reproduce, the relationship can become nonlinear due to the same reason that handling time makes functional response nonlinear. As such, the relationship between fecundity and number of prey eaten has many facets to be considered.

In addition to characterizing the relationship between foraging success and reproduction, it is important to characterize how individual variation in foraging success changes with the densities of interacting species. If the variation increases with the predator density, the mechanism described in this study may be considered as a general stabilizing factor. Although many functional response studies have characterized how prey density influences the average predation success, we know little about how the variance in predation success changes with the prey density because data are not analyzed in such a way. Recently, more functional response studies began to test the importance of the predator density (Kratina et al. 2009; Okuyama and Ruyle 2011; Okuyama 2012). Nevertheless, as discussed above, those data usually do not have the needed resolution because the standard protocol of these experiments is to count the number of prey consumed at the end of an experimental trial. However, given that empirical functional response studies continue to be numerous, if those experiments start collecting data at individual level (e.g., instead of recording *x* prey were consumed by *y* predators, record how many prey were consumed by each predator individuals), substantial information would accumulate to examine the mechanism described in this study.

Negative density dependence (e.g., Fig. 1B) is a well-recognized stabilizing factor, but mechanisms that produce negative density dependence are not well studied (McPeek 2012), which made it difficult to connect empirical studies and theoretical studies (but see Vadstein et al. 2012). This study proposed a general mechanism that can stabilize community dynamics and gave two explicit testable predictions (i.e., concave down relationship between reproduction and predation, and density-dependent individual variation in foraging success among predators). Even when functional response is apparently independent of the predator density, the mechanism described here still induces dynamics similar to predator-dependent functional responses. The effect is cryptic and has not received much attention because most studies focus on describing only mean responses. Describing individual variation in data will facilitate uncovering the potential cryptic predator dependence that may be generally present in many food webs.
